# Feasibility of OpenPose markerless motion analysis in a real athletics competition

**DOI:** 10.3389/fspor.2023.1298003

**Published:** 2024-01-05

**Authors:** Neil J. Cronin, Josh Walker, Catherine B. Tucker, Gareth Nicholson, Mark Cooke, Stéphane Merlino, Athanassios Bissas

**Affiliations:** ^1^ Neuromuscular Research Centre, Faculty of Sport and Health Sciences, University of Jyväskylä, Jyväskylä, Finland; ^2^ School of Education and Sciences, University of Gloucestershire, Gloucester, United Kingdom; ^3^ Carnegie School of Sport, Leeds Beckett University, Leeds, United Kingdom; ^4^ International Relations and Development Department, World Athletics, Monaco, Monaco

**Keywords:** motion capture, markerless tracking, artificial intelligence, kinematics, sprinting

## Abstract

This study tested the performance of OpenPose on footage collected by two cameras at 200 Hz from a real-life competitive setting by comparing it with manually analyzed data in SIMI motion. The same take-off recording from the men's Long Jump finals at the 2017 World Athletics Championships was used for both approaches (markerless and manual) to reconstruct the 3D coordinates from each of the camera's 2D coordinates. Joint angle and Centre of Mass (COM) variables during the final step and take-off phase of the jump were determined. Coefficients of Multiple Determinations (CMD) for joint angle waveforms showed large variation between athletes with the knee angle values typically being higher (take-off leg: 0.727 ± 0.242; swing leg: 0.729 ± 0.190) than those for hip (take-off leg: 0.388 ± 0.193; swing leg: 0.370 ± 0.227) and ankle angle (take-off leg: 0.247 ± 0.172; swing leg: 0.155 ± 0.228). COM data also showed considerable variation between athletes and parameters, with position (0.600 ± 0.322) and projection angle (0.658 ± 0.273) waveforms generally showing better agreement than COM velocity (0.217 ± 0.241). Agreement for discrete data was generally poor with high random error for joint kinematics and COM parameters at take-off and an average ICC across variables of 0.17. The poor agreement statistics and a range of unrealistic values returned by the pose estimation underline that OpenPose is not suitable for in-competition performance analysis in events such as the long jump, something that manual analysis still achieves with high levels of accuracy and reliability.

## 1 Introduction

Motion analysis is a common tool in sports biomechanics with expensive marker-based systems recognised as the gold standard technology when accuracy and speed of data processing are required. Marker-based technologies are often limited to laboratory conditions and require a comprehensive process of marker attachment. On the other hand, manual analysis of digital video images collected with visible light systems remains the most popular method for studying motion in competitive settings. This is because of inherently uncontrollable factors in the competition environment (weather, lighting, reflections, etc.), as well as the fact that marker-based systems cannot be used in such situations. Whilst digital cinematography does not necessarily require the attachment of markers to the object of interest (e.g., human body), it relies on a manual process of identifying key landmarks from each frame of the recording sequence, something that makes the whole process extremely time-consuming and prone to digitizing errors. Manual analysis requires an operator to digitize each key body landmark independently in each frame of a video capture, which is often at high frame rates (>100 Hz), and across two-or-more cameras for three-dimensional (3D) data. In cases where the full body needs to be analyzed, this means several key points [e.g., the commonly-used de Leva model (1) uses 17 points to compute centre of mass variables], something that further adds to the processing time. Furthermore, manual analysis is subject to human error, especially when joint centers such as the hip and shoulders are difficult to “visualize” and can often be occluded by other segments or even other athletes.

Notwithstanding the above challenges, manual analyses from competitive sport settings adhering to rigorous protocols have been providing the scientific and coaching communities with accurate and reliable kinematics and other performance metrics over the past few decades (2–7).

Recent developments in artificial intelligence (AI) have resulted in the proliferation of markerless methods, which have demonstrated reasonable accuracy in the study of human gait and various other movements (8–10). However, to date, markerless methods have mainly been applied in laboratory conditions, as they are often validated against optoelectronic motion analysis systems (9, 11–15). Whilst there are promising developments in sport-related markerless technologies, these still require the development of activity-specific models and multi-camera arrangements, which is difficult to achieve in competitive environments or diverse coaching settings (11, 12, 16, 17). This is also the case for open-source pose estimation algorithms, which, despite not being able to accurately track unusual body poses, are considered reasonably appropriate for single plane movements or coaching applications (10, 13, 18). For instance, in some cases, the combination of OpenPose with multiple synchronised video cameras has shown a mean accuracy of 30 mm or less for slow and relatively dynamic movements when compared against multiple camera optoelectronic systems (9). Whilst this level of accuracy could be acceptable for some applications, it still relies on a controlled environment and a large number of cameras. However, in other cases when only two cameras recording lower resolution images (smartphones) and the OpenCap (19) platform were used, the average RMSE across nine lower body angles was 5° with an average peak error of 10° (14).

It is essential to assess how well markerless models work in actual competition settings or outside of the laboratory. Would similar findings be observed when operating in a real-world setting with a limited number of cameras? This question needs exploring if markerless technologies are to become a credible, easy-to-use and cost-effective motion analysis for out-of-the lab applications. In this study we used a two-camera OpenPose markerless approach to examine 3D kinematics during the take-off of the men's Long Jump final from the 2017 World Championships and compared the results to manual analysis. As this was an initial attempt to test the performance of an open-source markerless method in a real-life competitive setting, we opted for an athletic movement that is highly dynamic yet performed primarily along a single plane of movement and governed by the same global constraints for all performers.

## 2 Materials and methods

### 2.1 Research approval

Data were collected as part of the 2017 World Athletics Championships Biomechanics Research Project, which took place at the London Stadium, United Kingdom. Use of the data for this study was approved by World Athletics (formerly the IAAF), who own and control the data, and locally by the institutional research ethics committee. Participants in this study provided written consent to take part. The study was conducted in accordance with the Declaration of Helsinki.

### 2.2 Data collection

Long Jump data were collected from the 12 finalists in the men's Long Jump competition at the 2017 World Athletics Championships (20). The jumps were recorded using two Sony PXW-FS5 high-speed video cameras (frame rate: 200 Hz, shutter speed: 1/1,750 s; ISO: 2,500; FHD: 1,920 × 1,080 pixels), which were used to capture the movement of the athletes as they approached and reached the take-off board. Cameras were placed approximately 25 m from the take-off board with the acute angle between the two optical axes at approximately 60°. Camera calibration was conducted before and after the competition using a rigid cuboid frame (3.044 m^3^) which was placed strategically around the take-off board and approach area. Each athlete's best jump (determined as their furthest legal jump) was used for further processing. As such, one file per athlete was analyzed.

#### 2.2.1 Manual tracking

Manual digitizing was conducted by a single, experienced operator in SIMI Motion (version 9.2.2, Simi Reality Motion Systems GmbH, Germany) to obtain kinematic data during the final step and take-off phase of the jump (from penultimate touchdown before the board to leaving the take-off board). Ten frames before and after this phase were also digitized to provide padding for subsequent filtering. An event synchronisation technique using a series of key events (touchdowns and toe-offs) was used to synchronize the two-dimensional (2D) coordinates from each camera. This was conducted by identifying initial contact with the take-off board in both cameras, and then moving forwards until toe-off. If the same frame of toe-off was identified in both cameras, this process was repeated for the penultimate ground contact. If all four key events were detected at the same frame in both cameras, they were deemed to be synchronized. In each file, the left and right shoulder, hip, knee, ankle, and metatarso-phalangeal joint centers were digitized frame-by-frame. Upon completion, adjustments were made using the points-over-frame method (21) to ensure consistency. The manual digitizing was carried out by an experienced operator (>1,000 h of digitizing) who used anatomical criteria alongside the software's features (e.g., zoom function) to identify the required body landmarks. This methodological approach has previously been shown to display high reliability in competition-based athletics settings (7, 22, 23). Reliability of the digitizing process was estimated by repeating the digitizing process with an intervening period of 48 h. The results showed a Root Mean Square Error (RMSE) across the three joint angles of 0.5^°^ with an Intraclass Correlation Coefficient (ICC) of 0.99, an RMSE for the height of Centre of Mass (COM) of 0.01 m with an ICC of 0.95, and an RMSE for COM velocity of 0.01 m/s with an ICC of 0.99. A Direct Linear Transformation algorithm (DLT) (24) was used to reconstruct the 3D coordinates from each camera's 2D x- and y-coordinates. The accuracy of the 3D reconstruction, measured as a percentage of the number of pixels in the image, was <1% each camera. In addition, a “central hip” marker was computed using the midpoint of the left and right hip joint centers (23), and used as a proxy of COM. Variable calculation was conducted in MATLAB (v2019b, MathWorks, Inc., MA), where hip, knee, and ankle joints were defined as the angle between the vectors of neighboring segments (e.g., the hip angle was defined as the angle between the shoulder-hip and hip-knee vectors on that side of the body). Manually digitized data were filtered using a 15 Hz low-pass, recursive, second-order Butterworth filter. The take-off leg was defined as the leg that contacts the take-off board, whilst the swing leg was the leg that does not contact the take-off board.

#### 2.2.2 Automatic tracking

Automatic tracking was carried out using OpenPose, an open-source method used for the detection of human body parts (25, 26), with the 25-keypoint model. Each camera view was analyzed separately. When tracking with OpenPose, additional people are often observed (e.g., competition judges or other athletes in the background) and the algorithm is not able to identify specific individuals consistently. To ensure that the intended athlete was selected, a semi-automated program was written in MATLAB to allow the selection of the target individual when necessary. The 2D coordinates predicted by OpenPose were firstly filtered using a median filter to gap-fill any missing frames, then smoothed with a 15 Hz low-pass filter, before being cropped to the same phase of the jump as the manually digitized data. The same DLT was also performed as for the manually digitized data, and joint angles were computed in the same way. The keypoint between the hips was used in the same way as the computed central hip marker described above, which was used as a proxy for COM.

### 2.3 Statistics

All statistical analyses were conducted in MATLAB and SPSS (version 28, IBM, NY). Agreement between methods for time-series joint angle and COM data was assessed with a coefficient of multiple determination (CMD) using the in-built MATLAB function “fitlm”, as used previously to display reliability of kinematic data (27). CMD quantifies the waveform similarity between two curves, with values ranging between zero (highly dissimilar waveforms) and one (highly similar waveforms). Therefore, although they account for similarity in curve shape, they do not consider the amplitude of the waveform. Comparisons between the two techniques for discrete (zero-dimensional) data, such as joint range of motion, joint angle minima and maxima, and COM take-off characteristics, was performed as follows: Limits of Agreement (LOA) incorporating Bias and Random Error were constructed to assess agreement between the two methods; RMSE was used to provide a level of accuracy for the OpenPose method; Intraclass correlation coefficients (ICC_3,1_) were calculated to provide a measure of relative reproducibility between the two techniques. CMD and ICC_3,1_ values were interpreted as: 0.00–0.50 = “poor”; 0.50–0.75 = “moderate”; 0.75–0.90 = “good”; and 0.90–1.00 = “excellent” based on the guidelines of Koo and Li (28) for ICC interpretation.

## 3 Results

**Figure 1:**
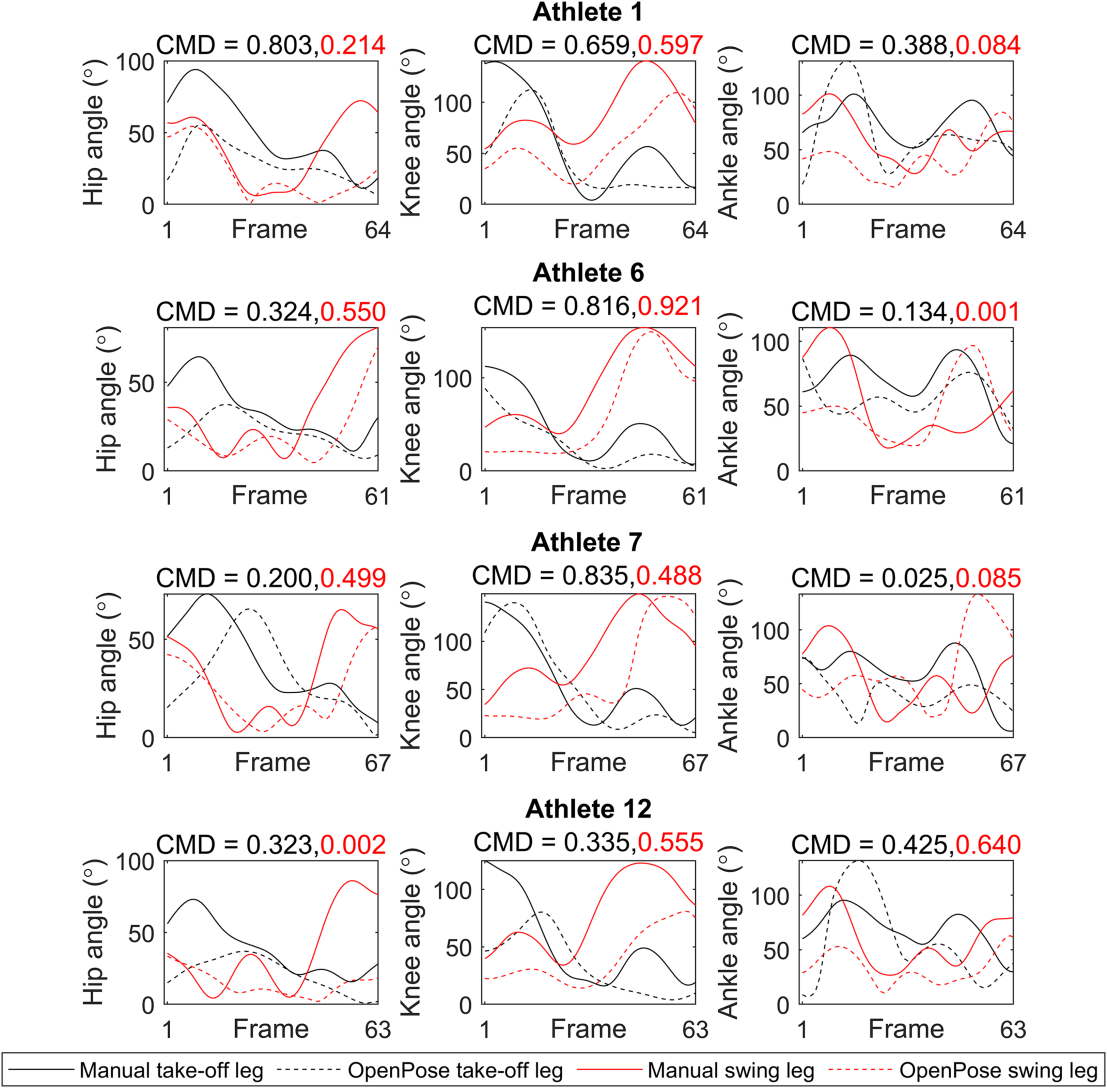
Joint angle waveforms (from penultimate touchdown before the board to leaving the take-off board) for four example athletes. Black lines represent the take-off leg and red lines represent swing leg, whilst solid lines represent data obtained with manual analysis and dashed lines represent data obtained with OpenPose. CMD values above each subplot show waveform similarity between methods for take-off leg (black numbers) and swing leg (red numbers). Data for the other eight athletes can be found in the Supplementary Material.

**Figure 2:**
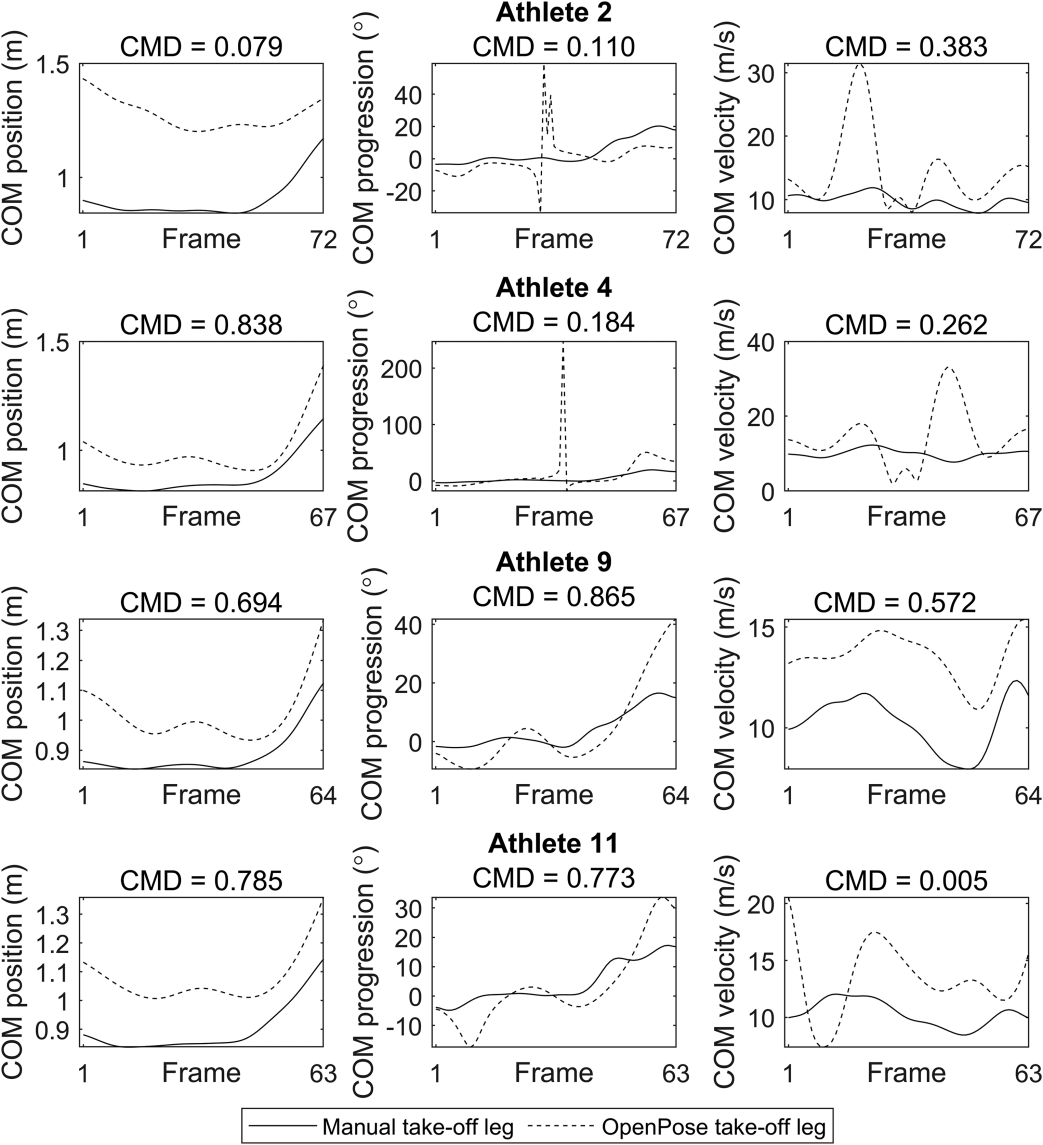
Position, progression angle, and velocity waveforms of the proxy COM markers (from penultimate touchdown before the board to leaving the take-off board) for four example athletes. Solid lines represent data obtained with manual analysis and dashed lines represent data obtained with OpenPose. CMD values above each subplot show waveform similarity between methods. Data for the other eight athletes can be found in the Supplementary Material.

CMD values for joint angle waveforms for the whole sequence showed large variation between athletes and between joints (Figure 1, Supplementary Material), with the knee angle CMD values typically being higher (take-off leg: 0.727 ± 0.242; swing leg: 0.729 ± 0.190) than those for hip angle (take-off leg: 0.388 ± 0.193; swing leg: 0.370 ± 0.227) and ankle angle (take-off leg: 0.247 ± 0.172; swing leg: 0.155 ± 0.228). Agreement between analysis techniques across all joint angles varied from moderate agreement for some athletes (e.g., Athlete 9: 0.516 ± 0.354) to poor for others (e.g., Athlete 2: 0.208 ± 0.122). CMD values for COM data also showed considerable variation between athletes and parameters (Figure 2, Supplementary Material), with COM position (0.600 ± 0.322) and projection angle (0.658 ± 0.273) waveforms generally showing better agreement than COM velocity (0.217 ± 0.241). Agreement between analysis techniques across COM parameters varied from moderate agreement for some athletes (e.g., Athlete 9: 0.710 ± 0.147) to poor for others (e.g., Athlete 2: 0.191 ± 0.167). Results concerning agreement between methods for discrete parameters are presented in Table 1. Agreement was generally poor with high random error for joint kinematics (ROM, minimum and maximum angles) and COM parameters at take-off (Table 1).

**Table 1:** Agreement statistics for discrete data between manual analysis and OpenPose methods.

	Bias	Random error	LOA	RMSE	ICC (3,1)
ROM TOL hip (°)	15.52	16.26	−0.74–31.78	17.43	0.21
ROM SWL hip (°)	20.89	29.97	−9.08–50.87	25.51	−0.05
ROM TOL knee (°)	15.66	31.41	−15.75–47.07	21.92	0.40
ROM SWL knee (°)	0.02	32.29	−32.28–32.31	15.78	0.78
ROM TOL ankle (°)	−15.22	75.51	−90.73–60.29	39.90	−0.31
ROM SWL ankle (°)	9.24	64.76	−55.52–74.00	32.96	0.01
Min TOL hip (°)	2.59	10.10	−7.51–12.68	5.57	−0.13
Min SWL hip (°)	−0.23	10.10	−10.32–9.87	4.94	0.27
Min TOL knee (°)	−1.09	14.90	−15.99–13.81	7.36	−0.32
Min SWL knee (°)	12.41	13.29	−0.88–25.69	14.00	0.14
Min TOL ankle (°)	−0.93	35.45	−36.38–34.51	17.34	−0.01
Min SWL ankle (°)	1.09	17.24	−16.15–18.32	8.49	0.54
Max TOL hip (°)	−0.35	14.91	−15.26–14.55	7.29	0.67
Max SWL hip (°)	3.86	26.20	−22.34–30.07	13.37	−0.15
Max TOL knee (°)	1.87	12.25	−10.38–14.12	6.27	0.85
Max SWL knee (°)	10.55	21.96	−11.41–32.50	15.04	0.66
Max TOL ankle (°)	−28.60	55.52	−84.12–26.91	39.42	0.06
Max SWL ankle (°)	10.93	65.92	−54.99–76.85	34.00	−0.15
Take-off COM angle (°)	−12.44	22.20	−34.63–9.76	16.50	0.00
Take-off COM height (m)	0.02	0.23	−0.22–0.25	0.11	0.19
Take-off COM velocity (m/s)	−4.74	2.83	−7.57–−1.91	4.94	0.00

LOA, limits of agreement; RMSE, root mean square error; ICC, intraclass correlation coefficient; ROM, range of motion; TOL, take-off leg; SWL, swing leg; COM, centre of mass.

## 4 Discussion

The aim of this study was to test the performance of an open-source markerless method on footage collected from a real-life competitive setting. We compared OpenPose with manually analyzed video data as this remains the best available method for obtaining 3D kinematics in a real competition setting where marker-based techniques are not realistic. The results regarding the suitability of OpenPose to track accurately and reliably a vigorous yet uncomplicated athletic movement were disappointing. This is demonstrated via both the discrete and continuous data generated by OpenPose with movement pattern curves vastly dissimilar to the criterion method, which as expected compared very well with past laboratory and field studies (4, 29, 30). The application of OpenPose yielded a spectrum of movement patterns so variable between and within athletes that it is impossible to identify a single variable or characteristic where OpenPose could be considered as a promising alternative to manual analysis. Even variables appearing to approximate graphically at times the manual data (e.g., left knee angle, COM spatial parameters) produced poor agreement statistics for discrete data against manual digitizing with total errors of such a large scale that prohibit any credible performance estimation. For instance, the random error for the range of motion for the swing leg's knee joint was 32.29°, an enormous variability for this type of measurement, something that consequently generated very large limits of agreement even with a bias of 0.02°. The impact of the lack of agreement between methods becomes more noticeable when popular and well-established take-off variables are used for the comparison. For example, the LOA for the COM take-off angle between the two methods were recorded as −34.63–9.76°. Considering for example that a minor increase in the take-off angle by 2° (from 20° to 22°) for a jump with a take-off velocity of 9.5 m/s and take-off height of 1.2 m increases the COM range from 8.27 m to 8.60 m, it becomes clear that such huge limits of agreement do not allow a valid quantification of the movement via the OpenPose tracking. Unsurprisingly, only two out of the 21 ICCs exceeded 0.75 with most of them exhibiting values around zero which alongside some negative ICCs demonstrates the absolute lack of correlation between the two methods and the extreme within-group variability for the OpenPose method. Overall, the quantities generated by OpenPose apart from diverging from those obtained by the manual analysis, reached unrealistic values (e.g., extreme COM velocities) which do not theoretically or practically correspond to the movement performed. These inaccuracies likely stem mainly from errors in pose estimation. OpenPose occasionally mistracks a body part, and even if this may happen for one key point, it can be sufficient to cause such large errors. We made no attempt to filter these out prior to running the DLT, since we wanted to test how well the method would work without human intervention in a difficult test setting where the cameras were positioned far from the motion.

OpenPose was selected in this study as one of the most popular open-source, ready-to-use, detection tools based on deep learning pose estimation with the acknowledgement that it was not designed to perform detailed biomechanical analyses. OpenPose is based on pre-determined landmarks (not exactly the same as the ones used in the manual analysis) and has been trained by non-experts, and whilst it draws information from an extensive library of labelled training images (26), these images are unlikely to reflect the intricacies of the long jump movement. Allowing the researcher to train their own models with self-selected landmarks and custom datasets (e.g., DeepLabCut) could be a potential advancement, however such techniques still present significant limitations and they do not necessarily perform better when compared to OpenPose for basic two-dimensional movements and 3D joint locations (10, 13, 18, 31, 32).

Despite the above limitations the motivation to invest in markerless motion analysis remains strong, as automated labelling of images will drastically reduce processing time and the need for a human operator to undertake such time-consuming process. However, it is expected that such developments will keep focusing on fixed laboratory settings or scenes where researchers can control spatial conditions. In contrast, for live competitions and settings beyond the control of the researcher (as in the current study), there is a long way to go for automated techniques which use the same pose estimation principles with OpenPose. This is mainly due to (a) the difficulty to place cameras in optimal positions, and (b) the level of accuracy needed for biomechanical outputs either for coaching or research purposes. Currently, manual analysis techniques based on 3D videography exhibit a very high degree of accuracy and reliability when analysing athletics competitions as our continuous research in the area has demonstrated (7, 22, 23, 33–35). Particularly, our accuracy measurements have varied between 0.2% and 1% RMSD for known 3D objects within the recorded space, whilst our digitising reliability (root-mean-square difference) for COM displacement remains consistently below 0.01 m, generally less than 1° for relative and absolute angles, and <0.02 m/s for segmental velocities. These standards backed by CMD scores of 0.99 for waveform similarity and ICCs approaching 1, have established the suitability of manual digitising for detailed biomechanical evaluation of athletic movement. That being said, these reliability metrics are based on a single operator (digitizer). One limitation of manual analysis is inter-operator agreement, a consideration not relevant to automated markerless motion analysis. However, most research studies which would employ manual analysis would use only one operator, so does not affect the overall purpose of this study.

The question that naturally arises is how markerless analysis can reach similar levels of precision in live out-of-the-lab settings. Currently and under controlled conditions, markerless (open-source or commercial) analysis is attaining on average error differences against multi-camera criterion methods far lower than in the current study for gait and jumping movements (e.g., ≤11° for joint angles, ≤2 m/s for COM horizontal velocities) (12–17, 36). Whilst these differences are perhaps acceptable for a general description of a movement, they are still considered large when subtle differences in performance are the focus of an investigation. With the error magnifying when measurements are conducted out of the laboratory, the additional challenge for markerless methodologies is to achieve high fidelity of movement reconstruction both in and out of the laboratory.

For out-of-the-lab applications, which is the focus of the current study, it is logical to argue that more cameras from multiple views would improve the markerless outcomes, however, this is not always feasible in a competition setting for various logistical and practical reasons. Increasing camera coverage would mean either placing more cameras in the stands or broadcasting platforms around the stadium, which is generally unrealistic due to the presence of TV crews and spectators. Similarly, adding cameras in the in-field could be a solution, but is problematic in any competition setting, especially athletics. These additional cameras could occlude television coverage, judges’ views, or other athletes’ performances. Thus, a core element of the current study was to use footage from only two cameras to compare manual and markerless analysis methods, as this provides a more representative comparison for competitive environments, unlike “validation” studies that use marker-based motion capture systems to compare with markerless motion capture.

The above logistical restrictions also apply to scenarios where the goal of markerless motion analysis is only to provide fast performance feedback to coaches and broadcasters during training and competitions with acknowledgement that data are not suitable for high precision biomechanical analysis. Markerless motion capture has been implemented in a field-based (albeit not competitive) setting in constrained movements such as baseball pitching yet with moderate agreement with marker-based systems [e.g., (37, 38)]. However, this is a setting where cameras can be placed in optimal locations whereas for competitive multi-spectator events, cameras are likely to be placed far away from the capture volume (e.g., in the stands or on broadcasting platforms), which means the movements must be recorded at long distances, and usually from suboptimal viewing angles (e.g., only one side covered).

A few future directions for automatic tracking during live competitions can be generated from this study. First, it may be necessary to identify biomechanical/performance variables that markerless approaches can provide with reasonable accuracy and reliability for different sports/events, acknowledging that these methods may not be able to compute all desired variables. This, however, necessitates an agreement between scientists and coaches of the acceptable level of accuracy that is required for different events and variables. Second, there is a need for sport-specific trained models (potentially even subject-specific) using data labelled by anatomical experts, rather than the typical crowd-sourcing approach used to train most open-access models. Even with the limitations outlined above, this may already yield substantial improvements in accuracy. Moreover, methodological refinements such as using inverse kinematics constrained models may help to improve tracking accuracy (16). Third, scientists will need to collaborate with sports event organizers such as federations, broadcasters, stadium owners, teams etc., in order to improve the general setup for performing markerless analysis with cameras. For scientists and practitioners, this, apart from allowing an optimal of cameras to be deployed, may provide solutions to issues such as calibrations constraints, occlusions from judges and TV crews, and poor/variable lighting effects. Improving the quality and speed of markerless analysis through set up, hardware and software improvements will make the end product appealing to industry stakeholders, in particular broadcasters who could incorporate fast data into their stream. This will also benefit scientists by drawing necessary funding to keep developing their applications.

## 5 Conclusions

This study selected one of the most popular open-source, ready-to-use, detection algorithms, based on deep learning pose estimation on pre-determined landmarks, to explore the feasibility of an off-the-shelf pose estimation algorithm in real athletics competitions. The results demonstrated emphatically that OpenPose in its current form is not suitable to track accurately and reliably a vigorous yet uncomplicated athletic movement such as long jump. The comparison with manual digitizing, which was employed as the criterion method, regarding both continuous and discrete data generated vastly dissimilar movement pattern curves and poor agreement statistics, with OpenPose data reaching unrealistic values for key performance variables which do not theoretically or practically correspond to the movement performed. Whilst markerless techniques are developing at an extremely fast rate, and it is expected that more focus will be placed on the out-of-the-lab applications in the near future, our findings suggest that OpenPose is not yet as accurate as manual analysis for obtaining reliable kinematic information about an athlete's in-competition performance.

## Data Availability

The datasets presented in this article are not readily available because the data is part of a wider project commissioned by World Athletics. Requests to access the datasets should be directed to AB, abissas@glos.ac.uk.
