# Supplementary figures and images for: Feasibility of OpenPose markerless motion analysis in a real athletics competition
Source: Front Sports Act Living. 2024 Jan 5;5:1298003. doi: 10.3389/fspor.2023.1298003 (PMC10796501; doi:10.3389/fspor.2023.1298003)

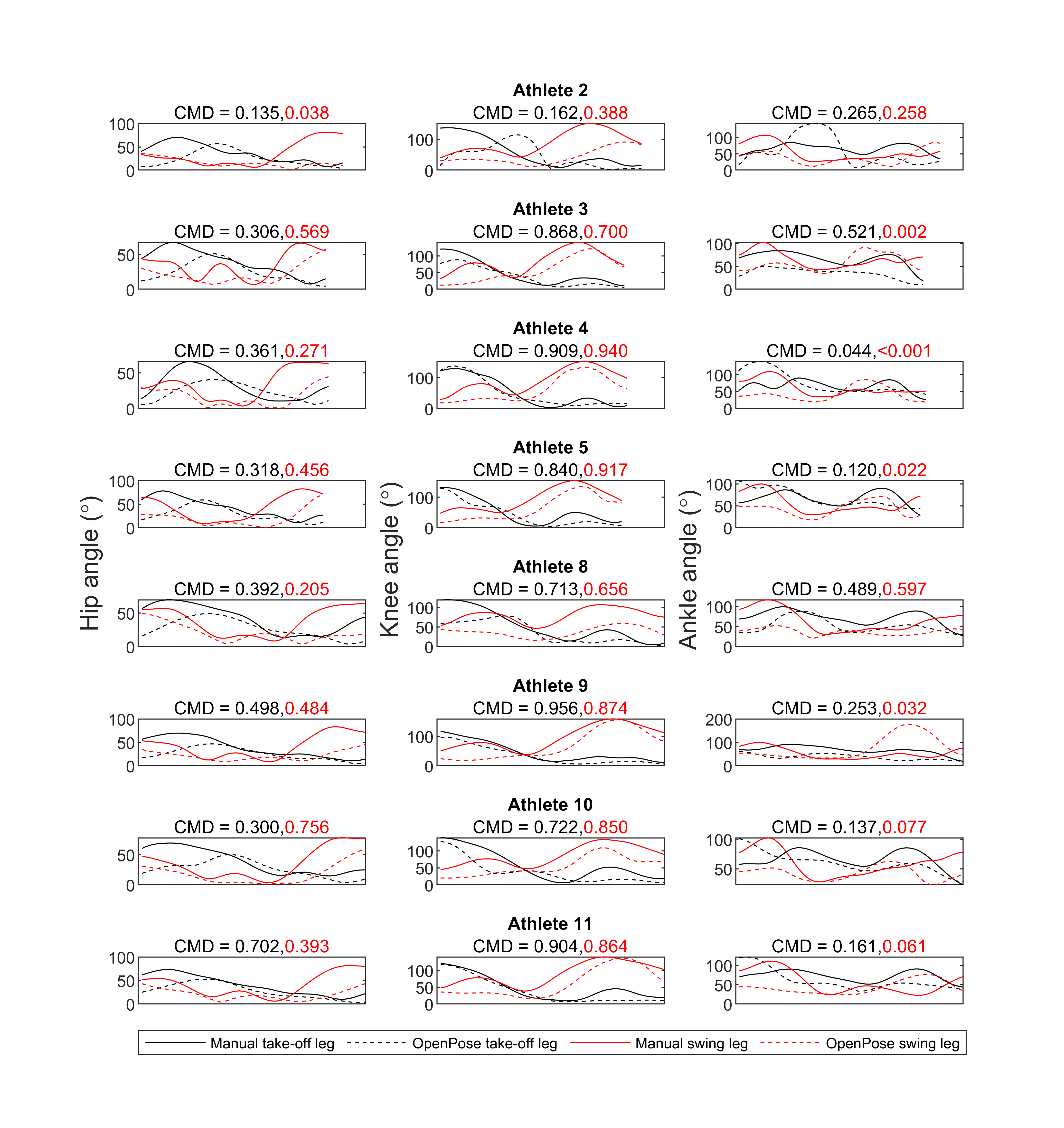

Supplement: Supplementary file 1 [file Image1.tif]

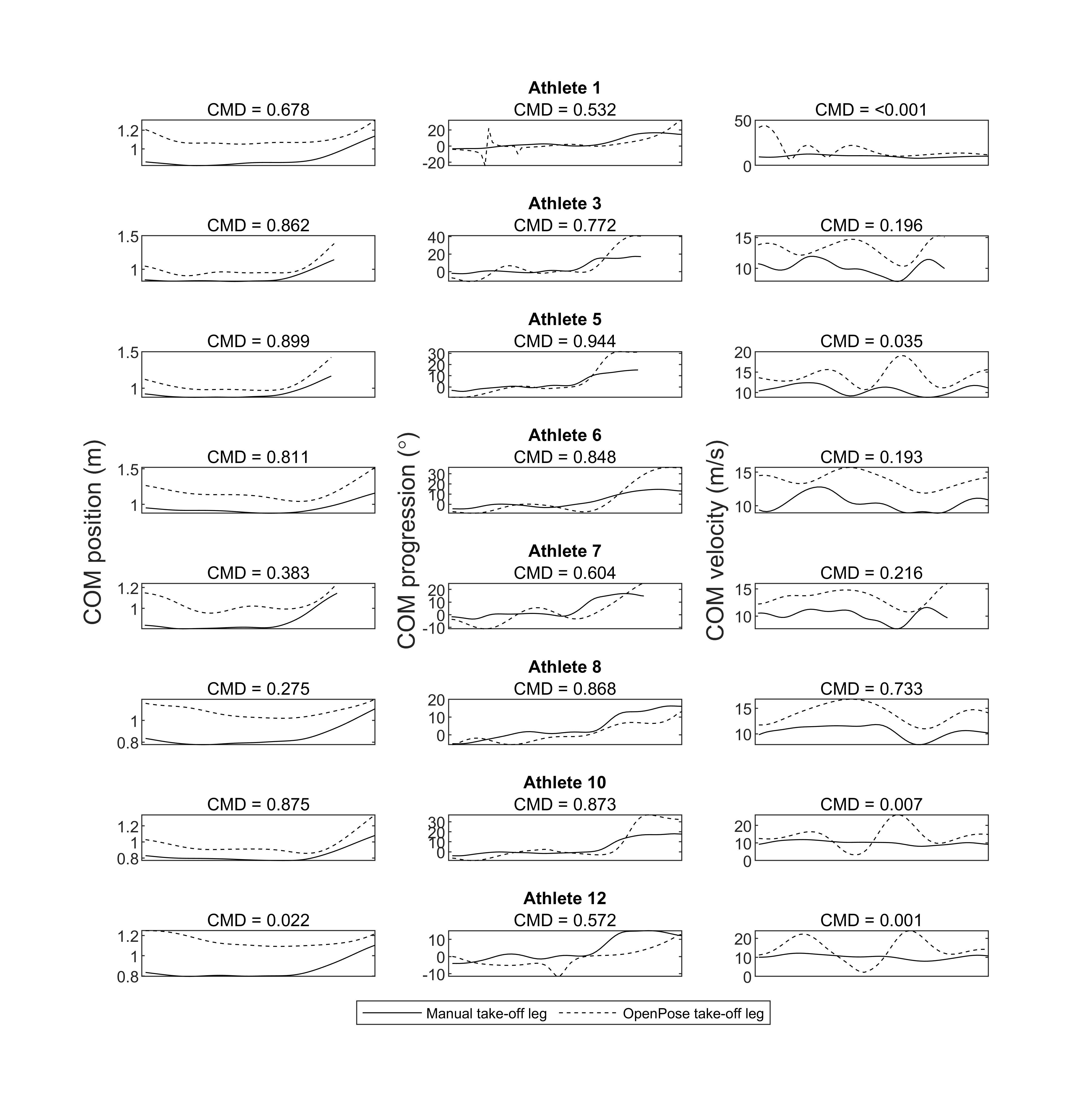

Supplement: Supplementary file 2 [file Image2.tif]
